# Tape-disc-loop-mediated isothermal amplification (TD-LAMP) method as noninvasive approach for diagnosis of cutaneous leishmaniasis caused by *L. tropica*

**DOI:** 10.1016/j.heliyon.2023.e21397

**Published:** 2023-10-29

**Authors:** Yasaman Taslimi, Sima Habibzadeh, Vahid Mashayekhi Goyonlo, Amin Akbarzadeh, Zahra Azarpour, Safoora Gharibzadeh, Mehrdad Shokouhy, Josefine Persson, Ali M. Harandi, Amir Mizbani, Sima Rafati

**Affiliations:** a Department of Immunotherapy and *Leishmania* Vaccine Research, Pasteur Institute of Iran, Tehran, Iran; b Cutaneous Leishmaniasis Research Center, Mashhad University of Medical Sciences, Mashhad, Iran; c Department of Epidemiology and Biostatistics, Pasteur Institute of Iran, Tehran, Iran; d Department of Microbiology and Immunology, Institute of Biomedicine, Sahlgrenska Academy, University of Gothenburg, Sweden; e Vaccine Evaluation Center, BC Children's Hospital Research Institute, The University of British Columbia, Vancouver, Canada; f ETH Zurich, Zurich, Switzerland

**Keywords:** Cutaneous leishmaniasis, Non-invasive skin sampling, Diagnosis, skin tape-disc, LAMP, Point of care, *L. tropica*

## Abstract

Cutaneous leishmaniasis (CL) is a parasitic disease caused by the bite of infectious female sand flies with high socioeconomic burdens. There is currently no non-invasive, point-of-care, diagnostic method with high sensitivity and specificity available for CL. We herein report the development of a non-invasive tape disc (TD) sampling method combined with a loop-mediated isothermal amplification (LAMP) assay using primer sets targeting kinetoplast DNA (kDNA) of *Leishmania tropica* (*L. tropica*) with a colorimetric readout for species-specific diagnosis of CL. We tested our Tape-Disc (TD)-LAMP method on a panel of skin samples collected by TD from 35 confirmed *L. tropica* patients, 35 healthy individuals and 35 patients with non-*L. tropica* infections. The detection limit of the TD-LAMP assay was determined as 1 fg (fg), and the assay sensitivity and specificity of 97 % and 100 % for *L. tropica* infection, respectively. This non-invasive, sensitive and rapid diagnostic method warrants further exploration of its use for differential diagnosis of CL in disease endemic settings.

## 1 Introduction

Leishmaniasis is a vector-borne disease caused by pathogenic parasites of the genus *Leishmania*, transmitted via the bite of different infected sand flies. Among 1000 species of sand flies, approximately hundreds of them are shown to be potential vectors for human leishmaniasis, including 26 New World *Lutzomyia* and 13 Old World *Phlebotomus* subgenera [1]. The three clinical forms of leishmaniases are cutaneous (CL), visceral (VL) and mucocutaneous (MCL). CL is mostly caused by *L. major*, *L. tropica*, *L. aethiopica* and rarely by *L. infantum* and *L*. *donovani* in the Old World, and by *L. mexicana*, *L. chagasi*, *L. amazonensis* and *L. braziliensis* in the New World [2]. The countries with high incidences of CL include Iran, Afghanistan, Brazil, Colombia, Costa Rica, Iraq, Peru, Sudan, Saudi Arabia, and Syria [3]. According to the WHO, approximately one billion people living in endemic areas are at risk of leishmaniosis, with an annual rate of one million for CL and 300,000 for VL [4].

Prompt and accurate diagnosis is of utmost importance for proper treatment and management of the disease. CL can be misdiagnosed due to the similarity of its clinical manifestations with cutaneous tuberculosis, fungi and bacterial infections, leprosy, eczema, and several other skin disorders [5]. There is currently no appropriate point-of-care (POC) diagnostics available for CL. Microscopic observation of parasites in the smears and *in vitro* culture of lesion exudates are the gold standard methods for diagnosis of leishmaniasis, even in well-equipped healthcare centers. These diagnostic methods have limitations such as misdiagnosis in low parasitemia conditions, the need for highly experienced staff for sample collection, preparation and visualization and time consuming [6]. In addition, there is a risk of bacterial and fungal contamination of the parasite cultures. Above all, these conventional methods are invasive, painful and may increase the risk of co-infection, especially in children.

Even though sensitive molecular biology tests such as PCR (polymerase chain reaction) have been used for CL diagnosis [[7], [8], [9]], they have not been widely accepted as an adequate replacement for the conventional methods for CL diagnoses due to the need for expensive equipment, reagents, experienced technicians, along with the sub-optimal specificity of the assays [10].

About two decades ago, a highly sensitive and specific detection technique called loop-mediated isothermal amplification (LAMP) was developed by Notomi et al. [11]. The reaction is performed in isothermal conditions and the result can be visible to the naked eye in less than 1 h, which makes the test very easy and practical for primary health care centers. LAMP has so far been used for the diagnosis of several diseases such as HBV, leishmaniasis and Covid-19 [[11], [12], [13], [14]].

There have been several studies on establishing proper non-invasive, POC, diagnostics for CL using microbiopsy tool for sampling which allowing fast frequently sampling without any pain, using swab for non-invasive sampling followed by PCR which will be useful for sensitive area of the body specially on the face [[15], [16], [17]]. Using cotton swabs for collecting the samples through wiping the ulcerative lesion and isolating the DNA by direct boil method followed by LAMP and species typing through user friendly portable sequencing called MinION ™ [18]. In other study the authors used Flinders Technology Associates (FTA) cards for noninvasive sampling followed by one step sensitive and fast LAMP assay for diagnosis of CL [19]. These non-invasive diagnosis methods were effective but were suitable only for wet ulcerative lesions. Therefore, more appropriate non-invasive sampling methods combined with POC diagnosis for both dry and wet forms of lesions are in high demand.

The ribosomal RNA internal transcribed spacer (ITS) and mitochondrial DNA minicircle network (kDNA) are among the target regions in PCR or LAMP for the diagnosis of leishmaniases [12]. In the present study, kDNA minicircles were selected due to their high copy number in *Leishmania*, which allows for higher sensitivity [9]. Further, previous studies used CsCl sedimentation methods followed by several time-consuming digestion steps for the isolation of kDNA minicircles [20,21]. In contrast, our proposed method is based on a simple approach for the isolation and enrichment of the kDNA minicircles from *L. tropica*.

We have recently reported a diagnostic method based on a non-invasive TD-based sampling followed by genomic DNA isolation and PCR-RFLP (Restriction fragment length polymorphism) [7]. Here, we have extended this work and developed a highly sensitive and specific TD-LAMP method based on an assay using primers for the kDNA minicircles of *L. tropica*. This report warrants further exploration on the usefulness of TD-LAMP for the diagnosis of CL infections caused by different *Leishmania* species.

## 2 Materials and methods

### 2.1 Ethics

The study was approved by the ethics committee of Pasteur institute of Iran under case number January 27, 2019, TP-9566. All the patients and healthy individuals or their guardians (in case of underage children) signed a written informed consent.

### 2.2 Clinical samples

**Fig. 1:**
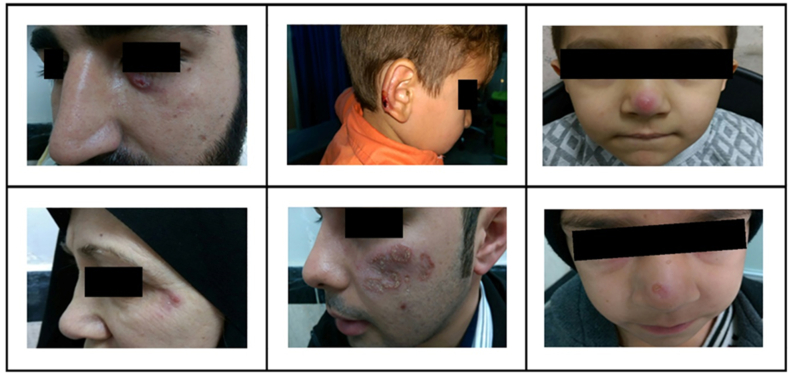
Representative skin lesions on sensitive areas of face of CL patients from which tape-disc samples were collected and subjected to the LAMP assay in the current study.

Samples were obtained by trained technicians using adhesive tape-discs (D-Squame, CuDerm Corporation, Texas, USA) from 35 healthy individuals and patients. Sampling was performed by placing a TD on the lesion followed by gently holding a plunger (D-Squame, CuDerm Corporation, Texas, USA) for 20 s on the TD in order to apply even pressure on the lesion parts. TDs were then stored at −20 °C until further use. The selected 35 patients had characteristic symptoms of CL and were positive for *Leishmania* infection confirmed by direct microscopy or culture. Skin lesions were located on different areas of the body such as sensitive areas of the face of CL patients from which tape-disc samples were collected and subjected to the LAMP assay in the current study (Fig. 1).

The samples were obtained from Abobargh health care center (Mashhad, North Khorasan province, Iran), located in a highly *L. tropica* endemic city, Mashhad. Samples were collected from 35 healthy individuals in the cities of Karaj and Tehran as non-endemic areas and 35 patients with cutaneous diseases other than CL. Within the latter cohort, 26 patients had diseases with clinical manifestations similar to CL, such as fungal infection (n = 14, *Aspergillus flavus*, *Aspergillus fumigatus*), bacterial infection (n = 6, *Escherichia coli*, *Klebsiella pneumoniae*, .*Enterococcus facalis, Staphylococcus saprophyticus*, *Pseudomonas aeroginosa*, *Staphylococcus aureus*), psoriasis plaque (n = 1), Malaria (n = 2, *Plasmodium falciparum*, *Plasmodium vivax*), toxoplasmosis (n = 1, *Toxoplasma gondii*), Human papillomavirus (n = 1), and Herpes simplex virus type 2 (n = 1). Furthermore, genomic DNA samples from two other causative agents of leishmaniases including *L. major* (MRHO/IR/75/ER) and *L. infantum* (MCAN/ES/98/LLM-877), together with samples from 7 confirmed *L. major* infected patients were included. The tape-discs were stored at −20 °C until further use. Patients′ samples genomic DNA was isolated, using DNAeasy Blood & Tissue kit (QIAGEN, Germany), from tape-discs containing lesions and normal skin samples as previously described [7] and used for both PCR-RFLP and LAMP as molecular diagnosis tests.

### 2.3 *Leishmania* PCR- RFLP

In order to confirm the infection on the 35 *L. tropica* and the seven *L. major* samples collected from patients, PCR-RFLP was performed as described elsewhere [7]. Briefly, the tape discs were cut into small pieces and incubated at 56 °C for 3 h in the lysis buffer containing proteinase k, and the next steps were performed according to the manufacturer's instruction (DNAeasy Blood & Tissue kit, Qiagen). The species of the *Leishmania* parasite in the samples was determined by amplification of the internal transcribed space 1 (ITS1) region of *Leishmania* using primers LITSR (5′-CTGGATCATTTTCCGATG-3′) and L5.8S (5′-TGATACCACTTATCGCACTT-3′). The reaction mixture contained 40 ng of each of the forward and reverse primers, 15 μl of 2x Taq PCR master mix (Pars tous, Iran) and 10 ng template DNA, in a total volume of 30 μl. The PCR program comprised (Eppendorf, Germany) an initial denaturation step of 5 min in 94 °C, followed by 35 cycles of 94 °C for 30 s, 53 °C for 1 min, and 72 °C for 1 min, followed by a final extension step at 72 °C for 15min. And the process continued followed by enzymatic digestion of the PCR product by *Hae* III (Takara Bio, USA).

### 2.4 Parasite culture and DNA purification from the reference strains

*L. tropica* (MOHM/IR/Khamesipour-Mashhad) promastigotes were cultured at 26 °C in Medium 199 (M199; Sigma, Germany) supplemented with 10 % heat-inactivated fetal calf serum (FCS; Gibco, Germany), 0.5 μg/ml Hemin (Sigma, Germany), 40 mM HEPES (Sigma, Germany), 0.1 mM Adenosine (Sigma, Germany), 1 mM l-glutamine (Sigma, Germany), and 100 μg/ml Gentamicin (Biosera, Spain). About 10^8^ parasites in the logarithmic phase of growth were washed three times with phosphate-buffered saline (PBS), and DNA was isolated using GenElute HP Plasmid Miniprep kit (Sigma, Germany) according to the manufacturer's instruction.

### 2.5 DNA sequencing

After DNA isolation, Plasmid safe ATP-Dependent DNase kit (Epicentre, USA) was used in order to selectively degrade linear DNA molecules, thereby enriching the sample with kDNA minicircles. A PCR purification kit (Jena Bioscience, Germany) was used to clean up the DNA samples. Finally, REPLI-g Mini kit (QIAGEN, Germany) was used for amplification of the circular DNA molecules before Next Generation Sequencing (NGS). The concentration and the quality of DNA were analyzed by using a Nanodrop™ spectrophotometer ND-1000. The Pacific BioSciences (PacBio, Sweden) NGS platform was used in order to obtain long reads comprising several repeats of each minicircle molecule, as generated by REPLI-g kit that enabled higher accuracy and obviated the need for assembly of short reads, which would be extremely challenging due to high similarity of minicircles’ DNA sequence.

### 2.6 TD-LAMP primer design

A set of novel primers specific for *L. tropica* (MOHM/IR/Khamesipour-Mashhad), including outer primers (F3, B3), inner primers (FIP, BIP) and loop primers (LF, LB), were designed by using online New England Bioscience (NEB) LAMP primer design tool, targeting the conserved regions of the kDNA minicircles. Sequences of LAMP primers are provided in Table 1.

**Table 1:** LAMP primers specifically targeting *L. tropica* kDNA minicircles.

Primer	Primer bp	Sequence
**FIP 1**	38	TCCTCTCGAAAGCGGCCTCCGCGCAGAGCAGAAACCTC
**BIP1**	39	CGGAGAAGCCCAATTCCAGGATGCCATTTTTGGCCTCGG
**F3 1**	19	AGAGACACAAAAGCCCCAG
**B3 1**	20	CCCCGTTCAAAAATCACCGA
**Loop F 1**	19	GGCTTGTTTTGGCGGTTTG
**Loop R 1**	17	CCACCCGGCCCTATTTT

### 2.7 Tape-disc-loop mediated isothermal amplification (TD-LAMP) test

Total DNA samples isolated from TD of *L. tropica* patients, healthy individuals and non-*L. tropica* diseases, and subjected to LAMP assay, using the primers as shown in Table 1. The LAMP reaction contained 40 pmol FIP and RIP, 20 pmol LF and LB, and 5 pmol F3 and B3 primers, in a total volume of 25 μl. The reaction also contained WarmStart Colorimetric LAMP 2X Master Mix (NEB, USA), an optimized formulation of *Bst* 2.0 DNA Polymerase in a low-buffer reaction solution consisting of a visible pH indicator, and 2 μl of DNA sample (about 10 ng DNA). In parallel, reactions using 1 ng DNA from the reference strains (*L. major* and *L. tropica*) and water were run in parallel as positive and negative controls, respectively. All reaction mixtures were incubated at 65 °C for 30 min in a heat block (TECHNE, USA). Visualization and analysis of TD-LAMP product was done by two methods; first, direct visual inspection, wherein a positive reaction would result in a color change of the LAMP reaction mixture to orange, while a negative sample would remain pink and second, the products were run on 1.5 % agarose gel containing ethidium bromide.

### 2.8 Determination of sensitivity and specificity of optimized TD-LAMP assay

In this study, the sensitivity of the TD-LAMP test was determined by using different concentrations of *L. tropica* genomic DNA, ranging from 10 ng down to 0.1 fg. The specificity of the test was evaluated by using the DNA isolated from different causative agents of human diseases such as *L. major*, *L. infantum*, *Plasmodium* spp (*falciparum*, *vivax*), Fungal spp (*Aspergillus flavus*, *Aspergillus fumigatus*)*, toxoplasma gondii*, bacterial *spp* (*Escherichia coli*, *Klebsiella pneumoniae, Enterococcus facalis, staphylococcus saprophyticus*, *pseudomonas aeroginosa*, *staphylococcus aureus*), Human papilloma virus and Herpes simplex virus type 2, one sample from a psoriasis plaque and seven *L. major* samples isolated from patients.

### 2.9 Sensitivity and specificity of the TD-LAMP assay in clinical samples

The sensitivity and specificity of TD-LAMP assay on clinical samples were assessed by using 35 positive *L. tropica* infected patients and 35 healthy individuals. Confirmation of these clinical samples was determined by microscopic evaluation, culture growth, and/or PCR-RFLP as the gold standard methods. The test sensitivity was calculated as (number of true positives)/(number of true positives + number of false negatives) × 100. Specificity was calculated as (number of true negatives)/(number of true negatives + number of false positives) × 100. The positive predictive value (PPV) was measured by (number of true positive)/(number of true positive + false positive) × 100, and the negative predictive value (NPV) was calculated by (number of true negative)/(number of true negative + false negative) × 100.

### 2.10 Statistical analysis

Performance of TD-LAMP test was evaluated by determining the sensitivity, specificity, PPV, and NPV values within their 95 % CI. Statistical analysis was performed using STATA 15.0 (StataCorp LLC, College Station, TX, USA).

## 3 Results

Tape-disc sampling method was used for sample collection from the skin of the study participants. Out of 35 *L. tropica* infected patients enrolled in this study, 24 (69 %) were female and the median age was 28 years (IQR: 4 to 43). Most of the lesions (85 %) were less than 3 cm in size, with a median duration of 4 months (IQR: 2–7 months). Lesions were located in the face (37 %), hand (37 %), foot (6 %), trunk (3 %), or other locations (17 %). The CL samples selected for this study were all confirmed positive by gold standard methods, i.e. direct microscopic detection of amastigotes, and parasitological evaluation of promastigotes and/or PCR-RFLP. Out of 35 healthy individuals, 26 (74 %) were female, and the median age was 45 years (IQR: 38 to 55), with no history of leishmaniasis (Table 2).

**Table 2:** Characteristic profile of *L. tropica* infected patients and healthy individuals enrolled in the study.

Type of samples	Age, median, year (IQR)	Sample size	Duration of lesion Median (IQR)	Lesion location %	Number of cases
**Lesion**	28 (IQR: 4, 43)	24 Female, 11 Male	4 (IQR: 2, 7)	Face: 37 Hand: 37 Foot: 6 Mix: 17	35
**Normal skin**	45 (IQR: 38, 55)	26 Female, 9 Male			35

### 3.1 Identification of *Leishmania* species in infected samples using TD-LAMP

**Fig. 2:**
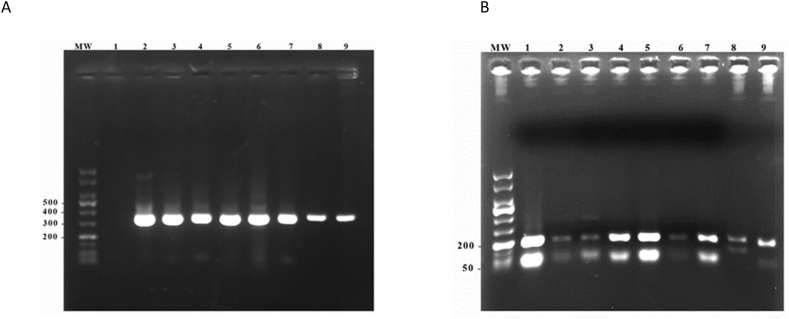
Agarose gel electrophoresis results of study participants using amplified ITS1 regions followed by RFLP. Panel A: H2O (lane 1), Patients samples (lane 2–7), *L. major* reference strain (lane 8) and *L. tropica* reference strain (lane 9). Panel B: Shows the results of *Hae*III digestion. In both panels, MW represents a 50-bp DNA ladder. Samples 1–7 were isolated from CL patients infected with *L. tropica*.

**Fig. 3:**
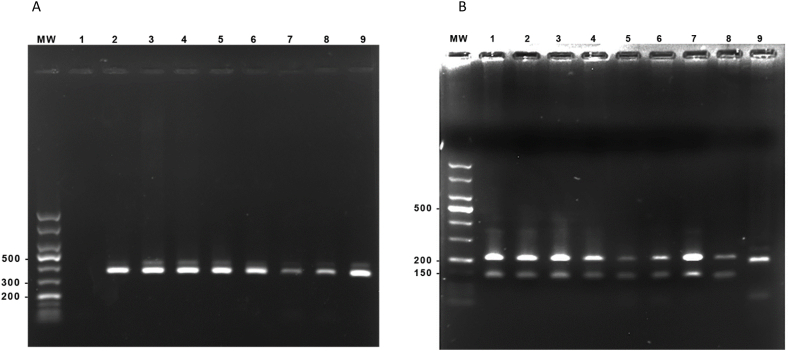
Results from agarose gel electrophoresis of amplified ITS1 regions followed by RFLP in samples collected from CL patients. Panel A: H2O (lane 1), Patients samples (lane 2–7), *L. major* reference strain (lane 8) and *L. tropica* reference strain (lane 9). Panel B: Shows the results of *Hae*III digestion. In both panels, MW represents a 50-bp DNA ladder. Samples 1–7 were isolated from CL patients infected with *L. major*.

In order to evaluate the performance of TD-LAMP in the diagnosis of *L. tropica* infection, 35 patients with a clinical appearance of CL were selected by a dermatologist and non-invasively sampled using adhesive TD. The DNA samples were analyzed by PCR test targeting the ITS-1, followed by running the reaction product on 1 % agarose gel. All 35 samples as well as the reference *L. major* and *L. tropica* genomic DNA had a 300-bp amplicon, which confirmed the presence of *Leishmania* DNA (Fig. 2A). The restriction fragments were visualized on 1.5 % agarose gels; the fragment sizes were 190 bps and 50 bps for *L. tropica*, and 210 bps and 160 bps for *L. major* (Fig. 2B). In parallel, seven previously collected samples from *L. major*-infected patients were re-confirmed by PCR (Fig. 3A) and RFLP tests (Fig. 3B).

### 3.2 TD-LAMP sensitivity and specificity

**Fig. 4:**
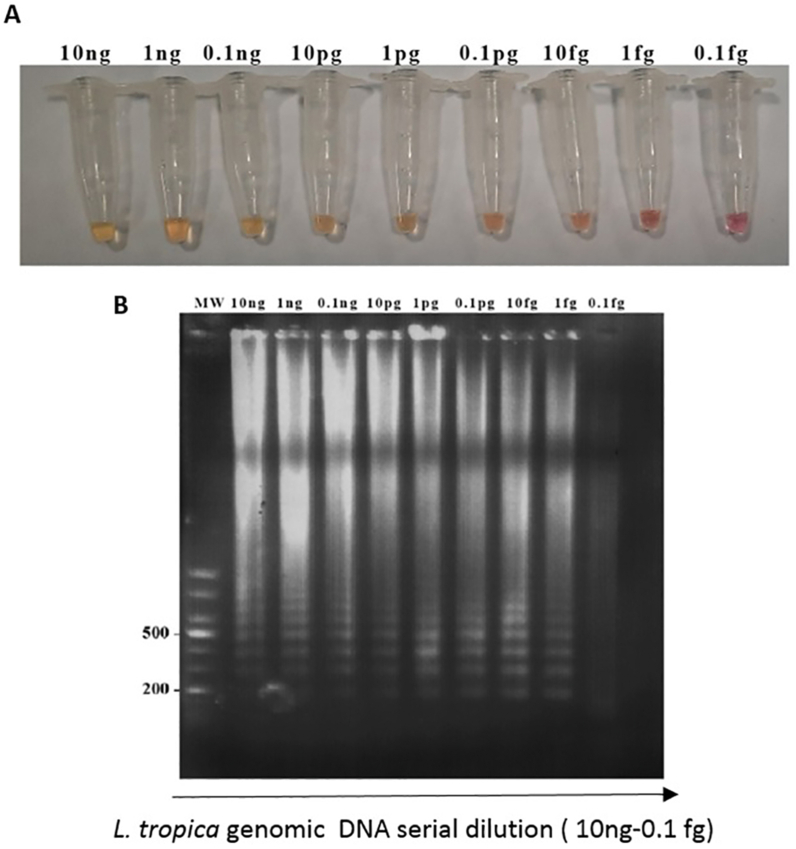
Determination of the sensitivity of the TD-LAMP test by using serial dilutions of *L. tropica* genomic DNA. Panel A: Detection of TD-LAMP products by color changing, tubes 1) 10 ng, 2) 1 ng, 3) 0.1 ng, 4) 10 pg, 5)1 pg, 6)0.1 pg 7)10 fg, 8) 1 fg, 9) 0.1 fg. Negative samples remained pink and positive ones turned into orange. Panel B: Agarose gel electrophoresis visualization of the TD-LAMP product.

To evaluate the sensitivity of the colorimetric TD-LAMP test, different amounts of *L. tropica* genomic DNA ranging from 10 ng down to 0.1 fg were used in each reaction. Successful DNA amplification was visually detectable with as low as 1 fg DNA after 30 min of incubation at 65 °C. All serial dilutions of *L. tropica* genomic DNA ranging from 10 ng to 1 fg showed a positive reaction color (orange) in the LAMP vials (Fig. 4A), and the expected pattern of ladder-shaped bands were observed on a 1.5 % agarose gel (Fig. 4B).

**Fig. 5:**
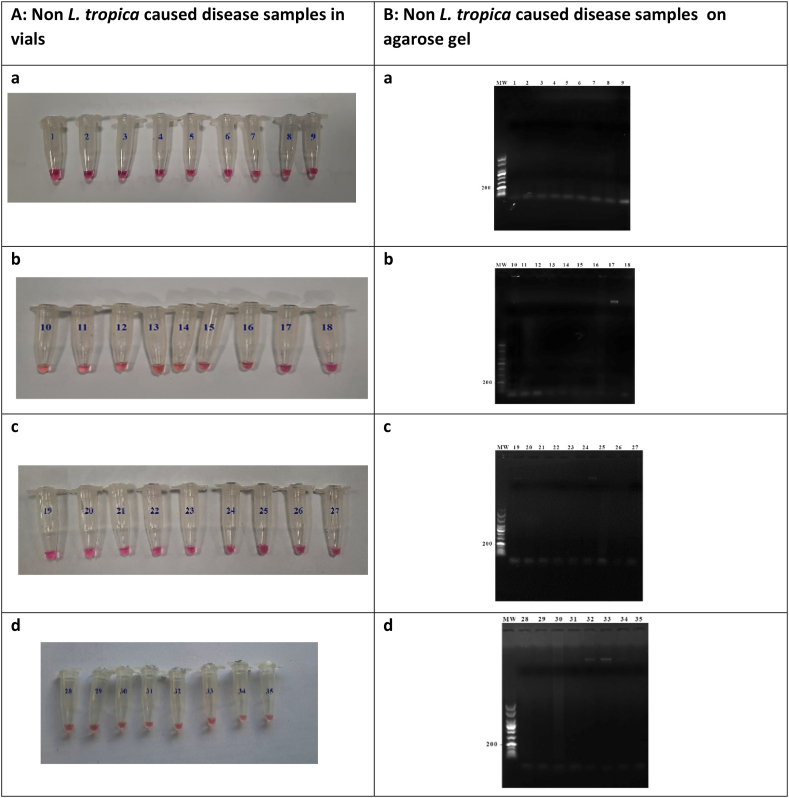
Determination of the specificity of the TD-LAMP. Specificity of TD-LAMP assay was determined by using genomic DNA from different non-leishmanial diseases, *L. major* infected patients, in addition to *L. infantum* and *L. major* p. Panel A: Visualization of TD-LAMP reaction tubes by naked eye. Panel B: agarose gel electrophoresis of TD-LAMP reaction products. The negative samples remained pink. The 50-bp DNA ladder was run in all gels. a) Lanes and vials 1–7 are *L. major* infected patients' DNA, lane and vial 8 is *L. infantum*, lane and vial 9 is *L. major* b) Lanes and vials 10–18 fungal genomics (*Aspergillus flavus*) c) Vials and lanes 19–23 for the remained fungal DNA (*Aspergillus fumigatus*), 24–25 *Plasmodium falciparum* and *vivax* DNA, 26 psoriasis, 27 human papilloma virus d) Lanes 28–33 bacteria DNA (*Escherichia coli, Klebsiella pneumoniae*, *Enterococcus facalis, staphylococcus saprophyticus, pseudomonas aeroginosa, staphylococcus aureus*), lane 34 *toxoplasma gondii* and lane 35 herpes simplex virus type 2.

In order to determine the specificity of designed TD-LAMP primers, different DNA samples from non-leishmanial and leishmanial lesions were examined. The non-leishmanial samples included diseases with similar skin manifestations, such as fungal and bacterial infections and psoriasis. Other human diseases including malaria, toxoplasmosis, and viral infections, in addition to leishmanial samples of *L. major* and *L. infantum* were also used. Non-leishmanial samples as well as *L. major* and *L. infantum* (Fig. 5A) had no reactivity and remained pink, without any appearance of bands on agarose gel (Fig. 5B). Therefore, the specificity of 100 % was determined for our designed TD-LAMP test.

**Fig. 6:**
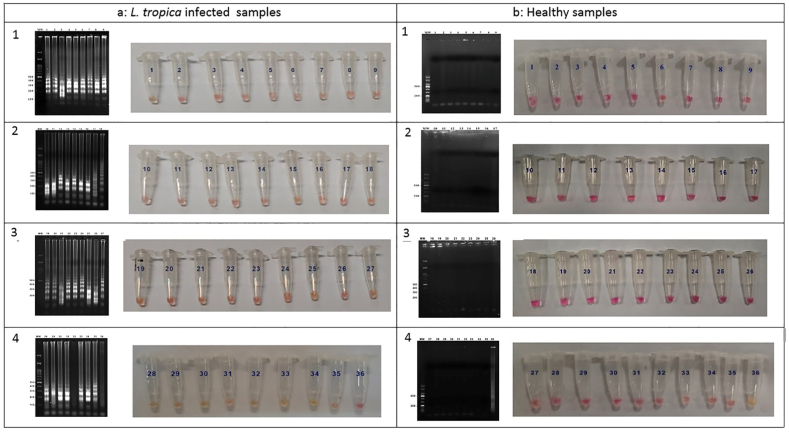
Colorimetric detection and visualization of TD-LAMP reaction on samples from *L. tropica* infected patients compared with healthy individuals. Fig. 6 part a shows the reaction vials and the agarose gel electrophoresis of 35 positive reaction of *L. tropica* infected patients except sample number 32 that was negative. Fig. 6 part b demonstrate the reaction vials together with agarose gel electrophoresis of the normal samples with no reactivity results. Sample 36 of part ”a” represents negative control (H2O) and part “b” the positive control (*L. tropica*).

Successful amplification reaction followed by colorimetric detection of *Leishmania* kDNA was achieved using LAMP in tubes containing DNA collected in a non-invasive manner. While the negative samples remain pink, the positive ones change color to green after only 30 min of incubation at 65 °C. For further confirmation, the LAMP products were run on 1.5 % agarose gels and visualized. Fig. 6a shows the infected *L. tropica* patients’ reaction vials and their corresponding agarose gel electrophoresis results. Fig. 6b indicates the vials together with agarose gels of the normal samples.

In summary, based on a total of 105 samples including 35 *L. tropica* confirmed CL patients, 35 healthy individuals and 35 non-*L. tropica* patient samples, the diagnostic sensitivity of our designed TD-LAMP test was 97 % (85.1%–99.9 %), the specificity was 100 % (94.9%–100 %), the positive predictive value (PPV) was 100 % (89.7%–100 %), and the negative predictive value was 98 % (92.4%–100 %), as shown in Table 3. The Receiver Operating Characteristic (ROC) area was 0.99 % (0.96%–1%). The combination of TD sampling and colorimetric LAMP test reported herein provides a highly specific and sensitive method for the diagnosis of CL caused by *L. tropica*.

**Table 3:** Different parameters of TD-LAMP including sensitivity, specificity, PPV and NPV.

N = 105	
a**Sensitivity**	**97 %**
**Specificity**	**100 %**
**PPV**	**100 %**
**NPV**	**98 %**

a The interpreter agreement between Disc and the gold standard using the Kappa statistic was calculated. The results showed that the agreement between the two methods is 99 %, which proves a level of agreement higher than expected by chance."

## 4 Discussion

Among neglected infected diseases, cutaneous Leishmaniasis is highly endemic in several countries. It is crucial to diagnose the disease in order to start treatment at the proper time to reduce the stigma of patients’ lesion and the risk of transmission. Unfortunately, in most leishmaniasis endemic countries the infrastructure available at healthcare centers is limited in terms of trained healthcare staff and modern instruments.

CL causes dermal lesions, ranging from small self-healing nodules to large non-healed ulcerative lesions. These manifestations sometimes overlap with other diseases, such as cutaneous tuberculosis, some fungal and bacterial infections, leprosy, and many other diseases that could potentially mislead the medical team in diagnosis, treatment and also epidemiological evaluations [5]. Therefore, a rapid, accurate, easy and noninvasive CL diagnostic method is of upmost importance for controlling the infection, selecting proper therapy and preventing wound disfiguration and stigma. Microscopic examination of lesion aspirations for visualization of *Leishmania* parasites is the gold standard diagnosis for CL; however, this method is time-consuming and requires a relatively high number of parasites in the lesion [22]. In our previous study, we investigated the possibility of establishing a non-invasive sampling method for CL diagnosis to reduce the risk of co-infection, pain and obviate the need for expert technicians. We used genomic DNA isolated from CL lesions for PCR followed by RFLP analysis for species identification [7]. Similar to our study, other groups have also proposed different non-invasive sampling methods for CL, but only for ulcerative lesions and by using cytology brush, filter paper [23,24], or cotton swabs [16,25].

In the present study, we sought to enhance the sensitivity, specificity and speed of our test by using an isothermal molecular diagnosis method, thereby obviating the need for expensive PCR machines. LAMP reactions with six primers have been indicated to have high sensitivity and specificity for detecting different diseases, such as malaria [26], Covid-19 [27], and TB [28]. Moreover, LAMP test has met all seven conditions to be classified as a good diagnostic method according to the WHO guidelines, ASSURED: affordable, sensitive, specific, user-friendly, rapid and robust, equipment-free, and deliverable to end users [29]. For some diseases, such as TB, WHO has recommended using LAMP instead of conventional diagnosis methods [30]. In the present study, we developed, optimized and tested a point of care diagnostic TD-LAMP method for CL caused by *L. tropica* infection.

We examined our designed TD-LAMP assay on 35 confirmed *L. tropica* CL samples, and evaluated its sensitivity and specificity by testing it on different concentrations of *L. tropica* genomic DNA, using healthy individual and non-leishmanial diseases and two other causing cutaneous leishmaniasis species. One of our limitation in this study was using a commercial LAMP master mix containing the dye to make sure that our designed primers work well on TD isolated samples; however, we aim at setting up our test to make it compatible with ordinary LAMP reactions supplemented by the dye added separately at reaction time to be a cost effective POC test. It is worth mentioning that LAMP has been previously used for the diagnosis of leishmaniasis by colorimetric SYBR green I, AuNP precipitates, MG and fluorescent SYBR [[31], [32], [33], [34], [35], [36], [37], [38]] which were sensitive and specific for diagnosis of leishmaniasis which in future we will. Another limitation of our study was using commercial isolation kit for extracting DNA from tape-discs that must be replaced by homemade protocols to be applicable for low income regions.

The results provided herein demonstrate 97 % sensitivity and 100 % specificity by the power of detecting 1 fg of *Leishmania* DNA per reaction, which was determined by both the naked eye and gel electrophoresis.

Similar to another leishmaniasis LAMP diagnosis test [12], our test also showed good performance both with a simple endpoint pH-dependent direct visualization method and agarose gel electrophoresis. One of the most important pillars of POC diagnosis is time, by reducing the time of diagnosis, treatment will be done faster and recovery achieved sooner [39].The strength of our method comes from using a non-invasive sampling method, suitable for both dry and wet (ulcerative and non-ulcerative) forms of CL lesions, even in a sensitive area of the body, such as the face, and more importantly, the species-specificity of the test in less than 30 min. This approach opens the possibility of using non-invasive sampling, followed by a sensitive, specific and fast point of care diagnostic method for cutaneous leishmaniasis, which would be highly valuable, especially in low-income countries**.**

In conclusion, we have developed, optimized and evaluated a TD-LAMP test for diagnosis of CL caused by *L. tropica*, in a fast and non-invasive manner. Future work could involve more *L. tropica* patients as well as designing similar TD-LAMP tests for detecting other CL-causing species, such as *L. major*.

## Funding

This project was funded by the European Union's 10.13039/501100007601Horizon 2020 research and innovation program under the Marie Skłodowska Curie grant agreement N°778,298. YT was supported by a doctoral student grant from 10.13039/501100010679Pasteur Institute of Iran (grant ID TP-9566).

## CRediT authorship contribution statement

**Yasaman Taslimi:** Writing – original draft, Visualization, Validation, Methodology, Investigation, Formal analysis. **Sima Habibzadeh:** Methodology, Investigation. **Vahid Mashayekhi Goyonlo:** Supervision. **Amin Akbarzadeh:** Investigation. **Zahra Azarpour:** Investigation. **Safoora Gharibzadeh:** Supervision, Software, Formal analysis. **Mehrdad Shokouhy:** Investigation. **Josefine Persson:** Investigation. **Ali M. Harandi:** Writing – review & editing, Supervision, Resources, Project administration, Funding acquisition, Conceptualization. **Amir Mizbani:** Writing – review & editing, Supervision, Project administration, Conceptualization. **Sima Rafati:** Writing – review & editing, Supervision, Resources, Project administration, Funding acquisition, Conceptualization.

## Declaration of competing interest

We confirm that the manuscript was approved by all authors and declare no conflict of interest.
